# The CRISPR Associated Protein Cas4 Is a 5′ to 3′ DNA Exonuclease with an Iron-Sulfur Cluster

**DOI:** 10.1371/journal.pone.0047232

**Published:** 2012-10-08

**Authors:** Jing Zhang, Taciana Kasciukovic, Malcolm F. White

**Affiliations:** Biomedical Sciences Research Complex, University of St Andrews, St Andrews, Fife, United Kingdom; University of Massachusetts Medical School, United States of America

## Abstract

The Cas4 protein is one of the core CRISPR-associated (Cas) proteins implicated in the prokaryotic CRISPR system for antiviral defence. Cas4 is thought to play a role in the capture of new viral DNA sequences for incorporation into the host genome. No biochemical activity has been reported for Cas4, but it is predicted to include a RecB nuclease domain. We show here that Cas4 family proteins from the archaeon *Sulfolobus solfataricus* utilise four conserved cysteine residues to bind an iron-sulfur cluster in an arrangement reminiscent of the AddB nuclease of *Bacillus subtilis*. The Cas4 family protein Sso0001 is a 5′ to 3′ single stranded DNA exonuclease *in vitro* that is stalled by extrahelical DNA adducts. A role for Cas4 in DNA duplex strand resectioning to generate recombinogenic 3′ single stranded DNA overhangs is proposed. Comparison of the AddB structure with that of a related bacterial nuclease from *Eubacterium rectales* reveals that the iron-sulfur cluster can be replaced by a zinc ion without disrupting the protein structure, with implications for the evolution of iron-sulfur binding proteins.

## Introduction

The CRISPR (Clusters of Regularly interspaced Palindromic Repeats) system is a recently discovered prokaryotic immune system providing protection against infection by mobile elements, including viruses [1]. Immunity is acquired by the capture of short viral DNA sequences known as “protospacers”, which are incorporated into the host genome, flanked by CRISPR repeat sequences and subsequently termed “spacers”. The CRISPR array is transcribed and processed to generate short CRISPR RNAs (crRNAs) that are utilised by CRISPR-associated (Cas) proteins to detect and subsequently degrade invading viruses with cognate sequences. In archaea, both viral DNA [2], [3] and RNA [4], [5] can be targetted for cleavage. The spacer acquisition process is not understood at a mechanistic level, but requires the ubiquitous Cas1 and Cas2 proteins, which have DNA and RNA endonuclease activities, respectively [6], [7]. Frequently, the *cas*1 and *cas*2 genes are found in close proximity to the gene encoding the Cas4 protein, one of the original core Cas proteins defined by Jansen and colleagues [8]. Cas4 bears a clear relationship with the archaeal-specific Cas protein Csa1 [9], and the two genes are frequently found adjacent to one another. Both have a RecB nuclease domain and three absolutely conserved cysteine residues near the C-terminus [8], [9]. It has been suggested that Csa1 be renamed Cas4′ in light of their close relationship [10]. In the archaeon *Thermoproteus tenax*, Cas4 and Csa1 have been shown to associate physically with Cas1 and Cas2 [11], suggesting that these four proteins may work together in the spacer acquisition pathway. In some genomes, such as *Myxococcus xanthus*, the *cas*1 and *cas*4 genes are fused, further emphasizing their likely functional interaction [12].

**Figure 1:**
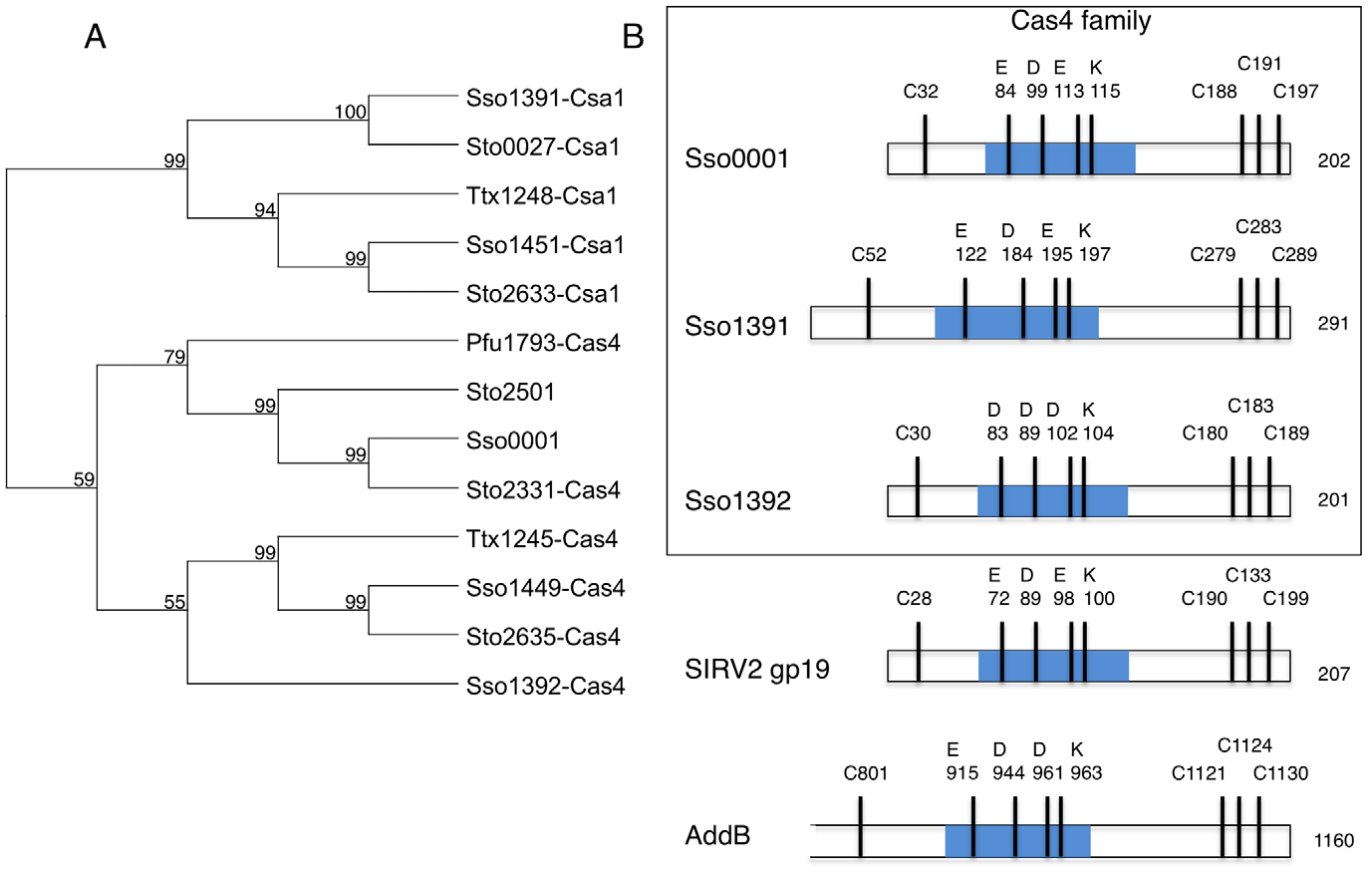
The Cas4 protein family. A. Unrooted bootstrapped phylogenetic tree showing a subset of Cas4 family proteins from archaea. Each protein is represented by a three-letter species code followed by the gene number from the respective genome sequences (Sso, *S. solfataricus*; Pfu, *P. furiosus*; Ttx, *T. tenax*). This neighbour-joining tree was generated from a T-coffee alignment of the proteins using MacVector, with pairwise distances between sequences uncorrected. The bootstrap values shown at each node represent the percentage of all trees (10000 total) agreeing with this topology. B. Cartoon showing the arrangement of the four cysteines acting as FeS ligands and the conserved residues contributing to the active site of the RecB nuclease, for the Cas4 family and related proteins. The blue shading denotes the central portion of the RecB-like domain in each protein.

Alignment of a subset of archaeal Cas4-family proteins from *S. solfataricus*, *Sulfolobus tokadaii*, *T. tenax* and *Pyrococcus furiosus*, followed by construction of a bootstrapped phylogenetic tree, demonstrates that the Csa1 and Cas4 proteins form sub-groups within the Cas4 family (Figure 1A). The *S. solfataricus* Sso0001 and *S. tokadaii* Sto2501 proteins, whose genes are not found near CRISPR loci, also group clearly within the Cas4 branch of the tree and each has the signature RecB domain and three conserved cysteine residues at the C-terminus.

In addition to the three C-terminal cysteines observed previously, there is a fourth conserved cysteine near the N-terminus of all Cas4 and Csa1 proteins (Figure 1B). This arrangement is strongly reminiscent of the AddB family of exonucleases implicated in DNA recombination in bacteria [13]. AddB utilises the four cysteine residues to form a conserved iron-sulfur cluster binding domain sometimes known as a “staple” that is essential for the structural integrity of the protein. A related protein, gp19 encoded by the archaeal virus SIRV2, shares the nuclease and four cysteine motifs and has recently been reported to possess Mg^2+^ dependent nuclease activity [14]. A cartoon representation of three representatives of the *S. solfataricus* Cas4 family together with the related nucleases AddB and SIRV2 gp19 is shown in Figure 1B. The conserved arrangement of the cysteines liganding the FeS cluster in AddB is apparent. Key active site residues corresponding to the RecB-type nuclease active site are also conserved.

Here, we report that two members of the Cas4 family from *S. solfataricus*, Sso0001 and Sso1391, are iron-sulfur proteins. We demonstrate that Sso0001 is a magnesium-dependent 5′-3′ ssDNA exonuclease *in vitro*. The role and evolution of iron-sulfur clusters in proteins and the potential function of the Cas4 nuclease in the CRISPR acquisition mechanism are discussed.

**Figure 2:**
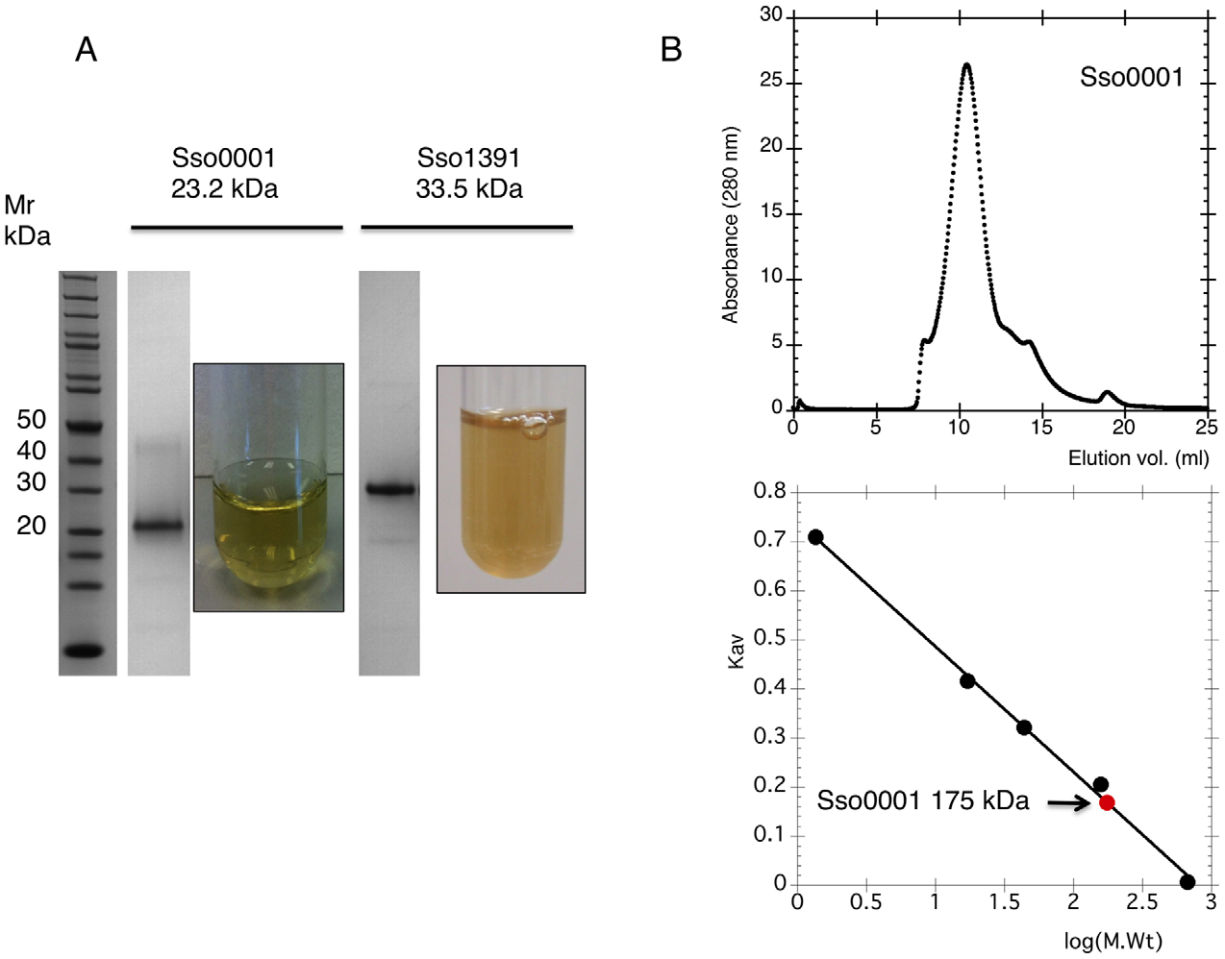
Cas4 family enzymes are Iron-Sulfur proteins. A. SDS-PAGE gel lanes showing purified Sso0001 and Sso1391 proteins. Concentrated samples of Sso0001 and Sso1391 had an olive-green appearance. B. The Sso0001 protein eluted from a calibrated superpose 12 column in one major symmetrical peak with a size corresponding to a molecular weight of 175 kDa.

## Materials and Methods

### Cloning, expression and purification of Sso0001 and Sso1391

The *sso*0001 gene was amplified from the *S. solfataricus* P2 genome using a forward primer 5′-TCATGTCATGATAACTGAATTTTTACTTAAAAAG and a reverse primer 5′-CCGCAAGCTTAGGTTAGTTTAGCTGGGC and cloned into pEHISTEV vector [15] at the *Nco*I and *Hind*III sites. The *sso*0001 D99A point mutation was generated by PCR mutagenesis using a forward primer 5′-GGAAGAGCCGCTGCAATAATTAGAAATG and a reverse primer 5′-CATTTCTAATTATTGCAGCGGCTCTTCC. The *sso*1391 gene was cloned into pDEST14 using the modified Gateway cloning system [16] with a forward gene specific primer: 5′-CCGAAAACCTGTATTTTCAGGGCATGTTCTTTACTCATTCAGATATG and a reverse gene specific primer: 5′-GGGGACCACTTTGTACAAGAAAGCTGGGTCCTAAGGGTGACAAACCTTATAAAAC.

**Figure 3:**
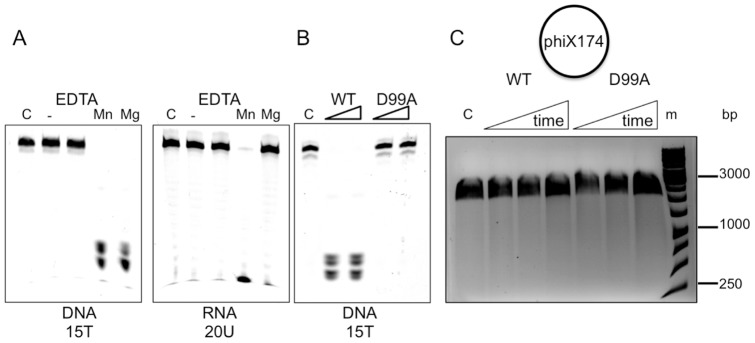
Cas4 family proteins are metal dependent nucleases. A. Sso0001 (0.6 µM) cleaves the ssDNA oligonucleotide 15T (1 µM) in the presence of either Mg^2+^ or Mn^2+^ but the ssRNA oligonucleotide 20U (1 µM) only in the presence of Mn^2+^. Both oligonucleotides were 5′-end labelled with a 5′-fluorescein moiety. Reactions were carried out at 75°C for 10 min. B. The Sso0001 D99A variant does not cleave ssDNA oligonucleotide 15T (1 µM) in the presence of Mg^2+^ ions. Assays were carried out as in panel A with 0.6 or 1.2 µM of the relevant protein. Reactions were carried out at 75°C for 10 min. C. Circular phiX174 virion ssDNA (60 nM) was incubated with 1.2 µM wild-type or 3 µM D99A Sso0001 for 10, 20 and 40 min at 55°C in the presence of MgCl_2_. No degradation of the DNA was observed, suggesting that Sso0001 requires a ssDNA end for activity. The control lane (C) was incubated in the same conditions in the absence of enzyme. dsDNA size markers (m) are indicated.

All proteins were expressed in *E. coli* Rosetta (DE3) pLysS. Cells were grown to A_600_  = 0.6 before induction with 0.4 mM IPTG at 37°C overnight. Cells were harvested by centrifugation at 4,000 rpm for 15 min.

**Figure 4:**
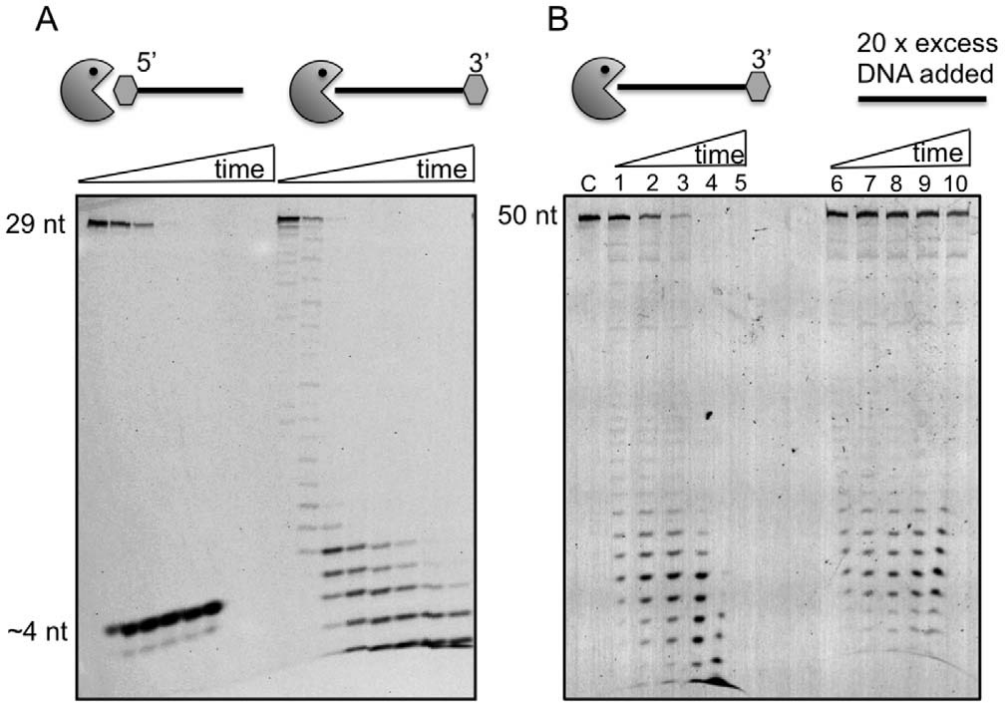
Cas4 family proteins are 5′-3′ exonucleases. A. Sso0001 (0.6 µM) cleaved the 5′ end labelled DNA 29mer (1 µM) in the presence of 10 mM Mg^2+^ into products with a size around 4 nt without any observable intermediates. In contrast, the 3′ end labelled DNA 29mer (1 µM) was cleaved to generate a ladder of progressively smaller products over the course of the reaction time in the presence of 10 mM Mg^2+^. Time points for both substrates were 0, 0.5, 1, 2, 3, 5, 10 and 15 min. The reaction temperature was 55°C. B. Sso0001 (0.3 µM) was incubated with 3′ end labelled DNA 50mer (500 nM) in reaction buffer at 55°C and products were analysed at 1, 2, 3, 5 and 10 min. In a parallel reaction (lanes 6–10), a 20 fold excess (10 µM) of unlabeled oligonucleotide of the same sequence was added to the reaction at the 1 min time point. A marked decrease in cleavage of the labelled oligonucleotide was observed, suggesting that Sso0001 is not highly processive, but can dissociate from substrates following each round of catalysis.

Sso0001 wild-type and the D99A variant proteins were purified in identical fashion. Cells were resuspended in buffer A (20 mM sodium phosphate pH 7.2, 500 mM NaCl) containing 10 mM imidazole, 100 µg/ml lysozyme and Complete EDTA-free protease inhibitors (Roche) and sonicated on ice for 5 cycles of 1 min with 3 min rest between cycles. The lysate was centrifuged at 25,000 rpm for 90 min at 4°C. The supernatant was filtered through 0.45 µm filters and then loaded onto a 5 ml HisTrap HP column (GE Healthcare) equilibrated in buffer A. After washing the column with 20 column volumes (CV) of buffer A containing 10 mM imidazole, bound proteins were eluted with a linear gradient from 10 to 600 mM imidazole. Fractions containing the protein were pooled, concentrated and loaded onto a HiPrep 16/60 Sephacryl S300 HR column (GE Healthcare) equilibrated in buffer B (20 mM Tris. HCl pH 7.5, 300 mM NaCl, 10% glycerol). Fractions containing the protein were pooled, concentrated and stored at −80°C.

**Figure 5:**
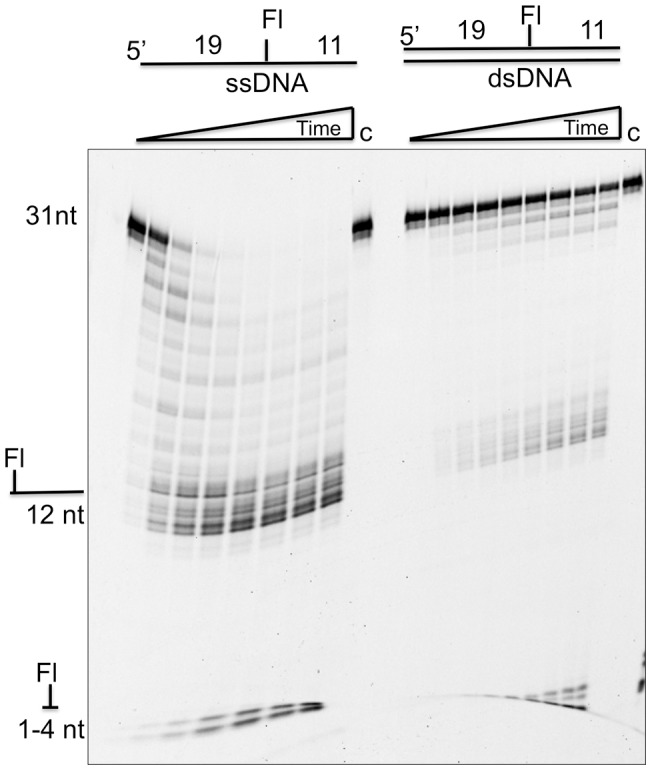
Cas4 family proteins are specific for single-stranded DNA and stalled by an internal extrahelical fluorescein. The left side of the gel shows degradation of a 31nt oligonucleotide with an internal fluorescein-dT at position 20. The right hand side shows this oligonucleotide in a 31bp DNA duplex (both at 1 µM final concentration). The assay was carried out with 0.6 µM Sso0001 at 55°C for time points of 0, 1, 2, 3, 5, 10, 20, 30 and 40 min in 10 mM Mg^2+^. Control lanes (c) lacked added Sso0001 enzyme.

Cells expressing Sso1391 were lysed by sonication as for Sso0001 with the addition of 10% glycerol to the lysis buffer. The supernatant was filtered and loaded onto a 5 ml IMAC FF column (GE Healthcare) pre-loaded with NiCl_2_ and equilibrated with buffer C (buffer A with 10% glycerol) with 10 mM imidazole. After washing the column with 10 CV of buffer C with 30 mM imidazole and 10 CV of buffer C with 50 mM imidazole, bound proteins were eluted in buffer C with 500 mM imidazole. Fractions containing the protein were dialysed in buffer D (50 mM Tris. HCl pH 7.5, 300 mM NaCl, 10% glycerol) and concentrated. Subsequently, the sample was loaded onto a HiPrep 16/60 Sephacryl S300 HR column equilibrated in buffer E (20 mM Tris. HCl pH 7.5, 250 mM NaCl, 10% glycerol). Fractions containing the protein were pooled, concentrated and stored at −80°C.

**Figure 6:**
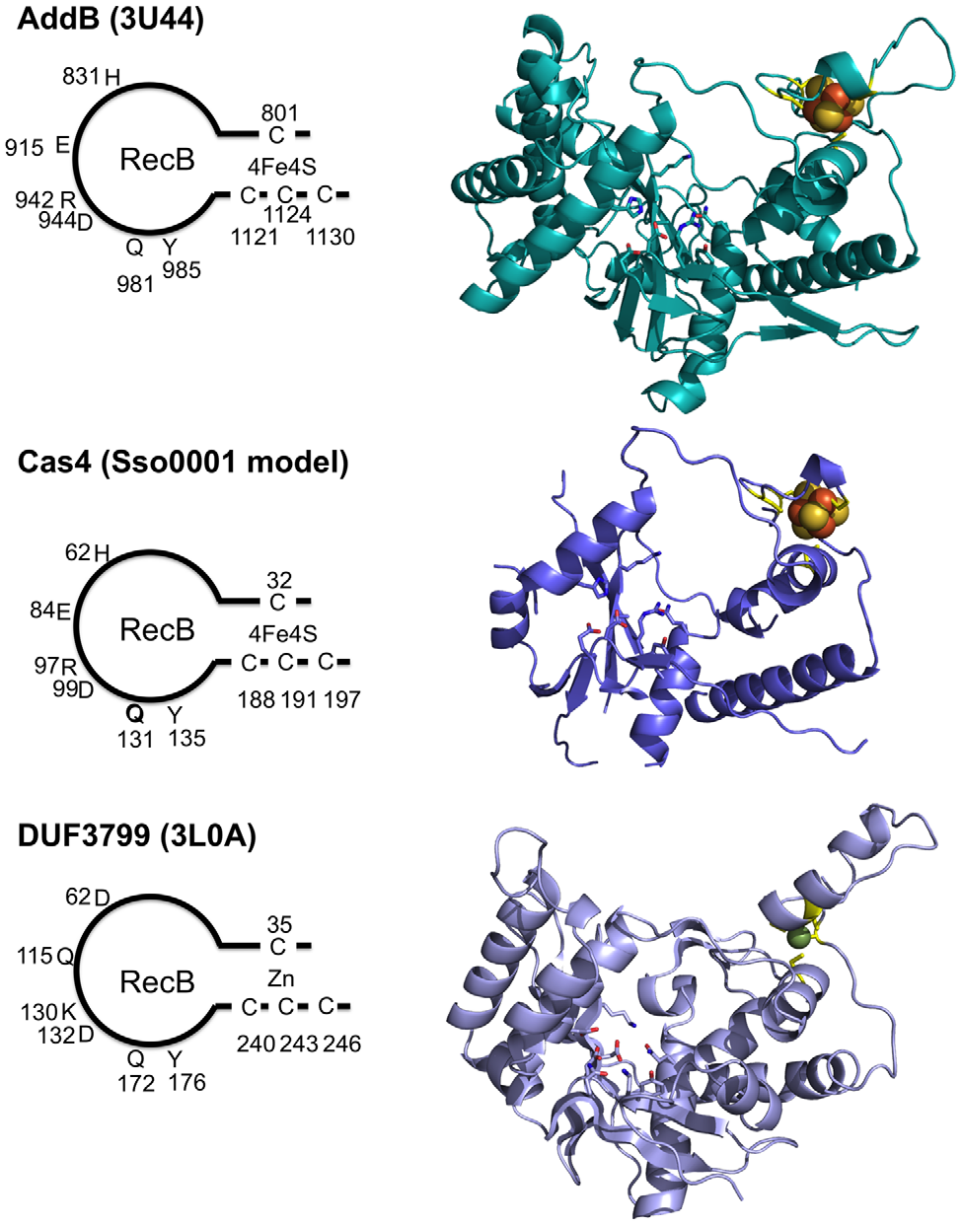
Structural similarities between AddB, Cas4 and a putative exonuclease DUF3799. All three structures show the same arrangement of four cysteine residues forming a metal ligand, coupled with a RecB nuclease domain. However, unlike AddB (PDB: 3U44) and Cas4, DUF3799 (PDB: 3L0A) has a zinc ion (green sphere) coordinated with the cysteines instead of iron. The cartoons on the left show the conserved residues implicated in nuclease activity and FeS cluster binding.

### Gel Filtration chromatography

A Superose-12 column (GE Healthcare) was calibrated using molecular weight standards (thyroglobulin, bovine gamma globulin, chicken ovalbumin, equine myoglobin and vitamin B12) in buffer E with a flow rate of 0.8 ml/min. The Sso0001 protein was analysed in the same conditions. The standards yielded a linear relationship for Kav to log molecular weight, which was used to calculate the native molecular weight of the Sso0001 protein [17].

**Figure 7:**
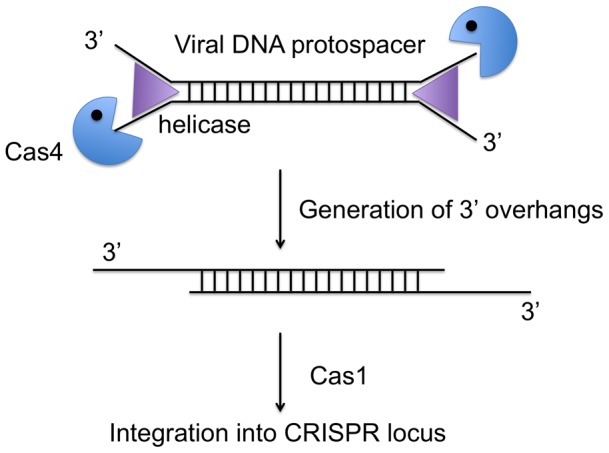
Possible role of Cas4 in the spacer acquisition pathway. In the spacer acquisition stage of the CRISPR system, the duplex viral DNA containing the protospacer may be unwound by the Cas3 helicase, providing substrates for Cas4. Processing by Cas4 would generate 3′ overhangs suitable for strand invasion at the CRISPR locus on the host chromosome.

### Nuclease activity of Sso0001

Purified Sso0001 wild-type protein or D99A variant was mixed with 1 µM oligonucleotide substrates in reaction buffer (20 mM MES pH 6.0, 10 mM DTT, 100 mM potassium glutamate, supplemented with 10 mM EDTA, 10 mM MgCl_2_ or 10 mM MnCl_2_ as indicated). The reaction was incubated at 55 or 75°C for the time indicated and quenched by the addition of EDTA to a final concentration of 20 mM. Samples were separated on 20% polyacrylamide, 7 M urea, 1× TBE gels as described previously [18]. Circular phiX174 virion DNA was purchased from New England Biolabs. Viron DNA (60 nM) was incubated with 1.2 µM wild-type or 3 µM D99A variant Sso0001 in reaction buffer supplemented with 10 mM MgCl_2_ and incubated for the times indicated at 55°C. On completion of the reaction the activity was quenched by the addition of EDTA to 20 mM final concentration and DNA was separated by electrophoresis on an agarose gel and visualised using ethidium bromide on a UV transilluminator.

For the competition assay, 300 nM Sso0001 was incubated with 500 nM oligonucleotide 3-Fl-50mer in reaction buffer at 55°C. After 1 min, 10 µM unlabelled oligonucleotide was added to the reaction. Reactions were processed as described above.

### Oligonucleotides

Oligonucleotides were chemically synthesised (Integrated DNA Technologies) incorporating a fluorescein (6FAM) label at a specific position (Fl).

5′-Fl-15T: 5′-Fl-TTTTTTTTTTTTTTT

5′-Fl-20U: 5′-Fl-UUUUUUUUUUUUUUUUUUUU

5′-Fl-29mer 5′-Fl-TTGTAGAGCAGTAAGAAGGTAATAGGAGT

3′-Fl-29mer 5′-TTGTAGAGCAGTAAGAAGGTAATAGGAGT-Fl

3-Fl-50mer: 5′- CCTCGAGGGATCCGTCCTAGCAAGCCGCTGCTACCGGAAGCTTCTGGACC-Fl

Nuclease substrate labelled with internal fluorescein-dT (Fl-dT):

Int-Fl-31mer 5′-CGATAAGCTTCTTCTTCTT(FI-dT)TCGGGTTTGGG

Duplex DNA with an internal fluorescein was generated by annealing the Int-Fl-31mer with its complementary strand:

5′-CCCAAACCCGAAAAGAAGAAGAAGCTTATCG.

### Iron Chelation Assay

Iron bound to Sso0001 was quantified by using the bathophenanthroline method [19]. 100 µl of 50 µM protein was mixed with 30 µl concentrated HCl and heated at 100°C for 15 min. The mixture was then centrifuged at 13,000×*g*. The supernatant was removed into a 2 ml tube and mixed with 1.3 ml 500 mM Tris-HCl pH 8.5. 100 µl of freshly prepared 5% ascorbic acid and 400 µl of 0.1% bathophenanthroline were then added to the tube and mixed thoroughly. The reaction was incubated at room temperature for 1 hour, after which the absorbance was measured. A standard curve consisting of known amounts of FeCl_2_ was used. The molar extinction coefficient for bathophenanthroline of 22,369 mol^−1^ cm^−1^ was used to calculate the iron concentration.

## Results and Discussion

### Cas4 family enzymes are Iron-Sulfur proteins

Genes for *sso*0001 and *sso*1391 were amplified by PCR from *S. solfataricus* chromosomal DNA and cloned into the pEHISTEV vector [15] as described in the methods. The proteins were expressed by inducing with IPTG and purified using Ni-affinity and gel filtration chromatography, yielding essentially pure protein (Figure 2). Both the Sso0001 and Sso1391 proteins had a olive-green colour characteristic of iron-sulfur proteins (Figure 2A). The Sso0001 protein was stable when stored at −80°C but lost colour and activity over several days when stored at 4°C, presumably due to degradation and loss of the FeS cluster. Sso1391 was markedly less stable than Sso0001, possibly due to the absence of Cas protein partners with which it may form a complex [20]. For Sso0001, the ratio of iron atoms in each protein molecule was estimated using an iron-chelation assay. Duplicate experiments yielded a mean ratio of 4.3 iron atoms per protein, consistent with the presence of a 4Fe:4S cluster. Individual substitution of each of the four cysteine residues of Sso0001 to alanine resulted in the expression of insoluble protein in *E. coli*, consistent with a role as Fe: S ligands, suggesting that the FeS cluster is crucial for the stability of the protein (data not shown).

Sso0001 was passed through a Superose 12 column calibrated with proteins of known size to allow an estimation of its quaternary structure. The retention time observed was consistent with a molecular weight of 175 kDa whereas a single subunit of Sso0001 has a molecular weight of 23 kDa (Figure 2B). Thus, Sso0001 is clearly not monomeric, although gel filtration does not allow molecular weights to be measured accurately. Many 5′ exonucleases assemble into toroidal structures that thread DNA through the central pore, including trimeric enzymes such as lambda exonuclease [21], [22] and the tetrameric RecE exonuclease [23], both of which are related to the Cas4 nuclease family.

### Sso0001 is a metal dependent nuclease

The nuclease activities of Sso0001 and Sso1391 were tested using fluorescent DNA or RNA oligonucleotides. No nuclease activity for Sso1391 was detected under any condition tested, probably because it forms a functional complex with Cas1 and Cas2 *in vivo* [11]. Cleavage of a 15T DNA oligonucleotide by Sso0001 was observed in the presence of either magnesium or manganese, yielding a cluster of products of around 1–5 nt in size (Figure 3A). A 20U RNA oligonucleotide was cleaved in the presence of manganese but not magnesium. RNA degradation was observed consistently to be slower than DNA degradation, suggesting that single-stranded DNA is the relevant target *in vivo*. To ensure that the nuclease activity was derived from the Sso0001 protein, a variant carrying the mutation D99A was constructed. This eliminates one of the essential metal binding ligands of the nuclease (Figure 1B); the equivalent mutation in the AddB nuclease abrogates nuclease activity [24]. The Sso0001 D99A variant failed to cut DNA (Figure 3B), confirming that the activity observed is due to the canonical RecB domain of the protein. To determine whether Sso0001 was an exo- or endo-nuclease, the enzyme was incubated with circular ssDNA from phiX174 (Figure 3C). No degradation of the DNA was observed over the time-course of the experiment, suggesting that Sso0001 is an exonuclease.

To characterize the exonuclease activity of Sso0001 in more detail, a DNA oligonucleotide substrate labelled at the 5′ or 3′ terminus with a 6FAM fluorescein moiety was utilized (Figure 4A). When labelled at the 5′ end, the only product observed had a size around 4 nt. In contrast, with the 3′-end labelled substrate, the progressive generation of smaller products differing in size by 1 nucleotide over the course of the reaction time was observed, finally resulting in fragments of 1–2 nt in size. This result demonstrates that Sso0001 acts as a 5′ to 3′ nuclease and appears to initiate cleavage at the 5′ end of the substrate. The reaction kinetics for the 5′- and 3′- labelled substrates were similar, suggesting that the 6FAM moiety at the 5′ end of the DNA, which has a six-carbon spacer, did not inhibit the enzyme significantly, although some effect on reaction rates cannot be ruled out. To determine whether Sso0001 was processive or distributive in its mode of action, the exonuclease reaction was initiated with a 50 nt 3′-FAM labelled substrate oligonucleotide (Figure 4B). After 1 min, a 20-fold molar excess of unlabeled substrate oligonucleotide was added and the reaction was allowed to proceed for 10 min. Comparison of the reaction products with matched control time points where no unlabeled substrate was added showed that substrates were not degraded to completion when excess unlabeled substrate was available. This suggests that Sso0001 is at least partly distributive rather than purely processive in its mode of action.

The activity of Sso0001 was further characterized by comparing the digestion of a 31 nt oligonucleotide containing an internal extra-helical fluorescein at position 19, in the context of both single-stranded and double-stranded DNA (Figure 5). The duplex DNA was clearly much more resistant to degradation, confirming the specificity of Sso0001 for ssDNA. The preference for ssDNA substrates is in marked contrast to the toroidal exonucleases such as lambda and RecE, which process dsDNA substrates [23]. A pronounced pause site was observed in the degradation of the internally labelled DNA, corresponding to the position of the extra-helical fluorescein 11 nt from the 3′ end. This confirms that the Cas4 family proteins are 5′-end directed nucleases and have difficulty bypassing bulky DNA adducts, probably because the ssDNA must be threaded through the active site of the enzyme to allow progressive cleavage in a 5′ to 3′ direction. Minor amounts of products of 1-4 nt size could be observed to accumulate over time, suggesting that the barrier presented by the extrahelical fluorescein was not absolute.

### Relationship of Cas4 with other nucleases

We have shown that the Cas4 protein belongs to a family of “iron staple” proteins. The best characterised is the AddB nuclease, a 5′-3′ exonuclease that functions along with the AddA helicase to generate recombination intermediates with 3′ ssDNA tails in *Bacillus subtilis* [13]. Other family members include Exonuclease V, a yeast mitochondrial enzyme that acts as a 5′-3′ ssDNA exonuclease during DNA replication [25] and the Dna2 nuclease/helicase involved in DNA replication and repair [26]. The structure of AddAB has been reported recently [27], revealing a helicase-nuclease machine with the C-terminal AddB nuclease domain positioned to cleave ssDNA generated by the helicase. The structure of the AddB domain can be used as a template to generate a structural model for Cas4 family proteins using the programme *Phyre* ^2^ [28]. A structural model for Sso0001 is shown alongside the AddB nuclease structure in Figure 6. Both structures have four cysteine residues clustered in a suitable configuration to bind a 4Fe:4S cluster. Conserved catalytic residues in the RecB nuclease domain are positioned in very similar locations and are likely to be involved in binding the catalytic metal ion(s) at the active site or have other roles in substrate binding or catalysis. Conserved residues are found at most equivalent positions in other Cas4 family members in *S. solfataricus* and in the SIRV2 viral nuclease protein (Figure 1B).

The structure of the AddB/Cas4 nuclease family has intriguing similarities to a family of putative nucleases found in bacteria and bacteriophages, typified by the protein EUBREC_2131 of *Eubacterium rectale,* a member of the DUF3799 protein family. Recently, a crystal structure of this protein (PDB code 3L0A) has been deposited in the protein data bank by a structural genomics consortium. Although as yet undescribed, the structure clearly shows the same arrangement of four cysteine residues forming a metal ligand, coupled with a RecB nuclease structure that is closely related to the RecE exonuclease of *E. coli* [23] (Figure 6). However, in this protein structure the FeS cluster has been replaced with a zinc ion. The role of iron-sulfur clusters in proteins such as AddB, as well as helicases and polymerases, is a matter of some debate (see [29] for a recent review). One school of thought is that these clusters function as redox sensors and may even be used actively to detect DNA damage [30]. Another hypothesis is that FeS clusters in many proteins are purely structural features, perhaps evolutionary relics of an anaerobic past when FeS clusters were much more common in proteins [31]. One prediction of the latter scenario is that FeS clusters may have been replaced gradually with cysteine-coordinated zinc ions in the course of evolution. The structural comparison of the AddB/Cas4 nuclease with the *E. rectale* nuclease may be a case in point, although one cannot rule out the possibility that the zinc ion in the *E. rectale* nuclease is artifactual until biochemical data are available to support its functional significance. Recent studies have shown that in eukaryotic DNA polymerases a four cysteine motif shown to bind zinc on overexpression in *E. coli* does in fact bind a 4Fe-4S cluster *in vivo* [32]. Likewise, other known FeS proteins, including Nar1 from *Saccharomyces cerevisiae* and IscU from *Haemophilius influenza*, have been found to contain zinc upon overexpression in *E. coli* [33], [34].

### Potential roles of Cas4 in the CRISPR system

The activity observed for the Sso0001 protein is strong evidence that Cas4 family proteins, like AddB, are all 5′–3′ DNA exonucleases *in vivo*. Since there is already evidence that Cas4 forms functional complexes with Cas1 and Cas2, it is likely that the nuclease activity of Cas4 is modulated and controlled by its partner proteins, ensuring that its potential for DNA degradation is kept under tight control. One possible role for Cas4 in the CRISPR acquisition pathway is to generate recombinogenic 3′-ssDNA overhangs in duplex DNA protospacers selected for incorporation into the genome (Figure 7). Although the mechanism of Cas1-mediated DNA capture is not understood, it may well require strand invasion of the new DNA and some form of recombination or integration event catalyzed by Cas1 [35]. Since Cas4-family exonucleases are active against ssDNA, end resectioning would require the activity of a helicase to unwind duplex DNA substrates. This could conceivably be the Cas3 helicase [36], or alternatively another cellular (non-Cas) helicase such as HerA, whose gene is sometimes found in association with genes encoding Cas proteins [10], might be recruited. In species lacking the *cas*4 gene an alternative end-resectioning nuclease could fulfill this function.

## Conclusions

In summary, we have demonstrated that the Cas4 family of proteins, implicated in the acquisition of new spacer sequences in the CRISPR system, are 5′ end directed 5′–3′ ssDNA exonucleases with an iron-sulfur cluster binding site. The FeS cluster is important for the overall stability of the protein, as has been observed for the related AddB nuclease [13]. The role of Cas4 *in vivo* may be to process viral DNA to generate new spacers with 3′ ssDNA overhangs suitable for recombination or integration in a CRISPR locus mediated by Cas1.
